# Editorial: Advances in Functional Neurosurgery

**DOI:** 10.3389/fneur.2021.812100

**Published:** 2021-12-17

**Authors:** Carlo Alberto Artusi, Adolfo Ramirez-Zamora, Marco Bozzali

**Affiliations:** ^1^Department of Neurosciences Rita Levi Montalcini, University of Turin, Turin, Italy; ^2^Department of Neurology, University of Florida, Gainesville, FL, United States

**Keywords:** functional neurosurgery, deep brain stimulation, Parkinson's disease, dystonia, glioma, electrocorticography, neuroimaging, movement disorders

In the last 20 years, there have been considerable advances in the techniques, technologies, and approaches devoted to functional neurosurgery. These advances have led to improved stereotactic accuracy, better outcomes, a lower rate of complications, and an increasing number of patients benefiting from this neurosurgical approach. In particular, Deep Brain Stimulation (DBS) has become the leading stereotactic technique for the treatment of refractory neurological symptoms in patients with Parkinson's disease (PD), tremor, and dystonia. Research outcomes in the field are often limited by small sample sizes, heterogeneity of participants, and lack of reliable neurophysiological biomarkers. However, the experience acquired and the technological innovations in the fields of surgery and neuroimaging have made new strategies available to improve the accuracy of the current practice.

In this Research Topic, a range of exciting advances in functional neurosurgery are showcased.

DBS targeting and technological innovations in implanted devices are among the most relevant challenges of current translational and clinical research, especially in movement disorders. In this Research Topic, Holanda et al. nicely reviewed a single center, 15-year experience with DBS of the Globus Pallidus pars interna in patients with PD and dystonia at the University of Florida, Fixel Institute for Neurological Diseases. The Authors analyzed the precise lead targeting in 253 patients with PD and 80 with dystonia and reported a progressive shift in the location of the stereotactic target in the PD group. At this center, the final location of DBS leads moved significantly more laterally, posteriorly, and dorsally over time. Conversely, the dystonia group did not show significant changes in lead position over time. These findings highlight two important aspects: (i) the importance of technical expertise in centers offering DBS, which might refine surgical techniques over time based on clinical experience; and (ii) the importance of a systematic post-operative DBS lead measurement protocol, which is essential to inform and optimize DBS programming. Consistent feedback from personal experience is indeed critical to improving DBS techniques and outcomes.

Other interesting findings published in this Research Topic relate to the importance of DBS targeting in patients with movement disorders. Prior reports suggest that weight gain is common in PD patients after subthalamic nucleus (STN)-DBS (1). Potential mechanisms include reduction in energy expenditure, changes in eating behavior and food intake, perturbations of homeostatic control, changes in hormone and neurotransmitter systems, and improvement in motor function. He et al. discuss their experience regarding weight changes in dystonia patients who underwent STN-DBS. They analyzed data from 54 patients with isolated dystonia who were treated with bilateral STN-DBS and observed that 78.7% of them exhibited post-operative weight gain, with a percentage of overweight or obese patients increasing from preoperative 25.5 to 48.9% after surgery. While confirming that subthalamic DBS is an effective treatment for isolated dystonia, they found for the first time that this treatment may cause post-operative weight gain, especially in females. Thus, centers offering DBS for dystonia should be careful in choosing STN as a therapeutic target for overweight or obese patients, especially women.

Over recent years, one of the main innovations in DBS devices has been the introduction of rechargeable Implantable Pulse Generators (IPGs). Qiu et al. published the results of a survey on preference and satisfaction of different IPGs among 220 PD patients treated with DBS in China. They found that the choice between rechargeable and non-rechargeable IPGs was significantly associated with affordability. Moreover, in China, the choice between domestic and international DBS device brands is available to patients. The survey showed that the preference between either device's brand was mainly driven by the affordability and the possibility of remote programming of stimulation settings.

Functional neurosurgery is not limited to DBS but is widely used for the neurosurgical treatment of various brain tumors. An example of new methods for improvement of surgical accuracy in patients with glioma is illustrated in an article by Kanaya et al. They reported the case of a patient with left temporal glioma, who was efficaciously operated on using an intraoperative passive language mapping through electrocorticographic (ECoG) investigation.

Other neurophysiological, imaging and technological advances are moving forward in the field of functional neurosurgery, and there is optimism about novel methods of analysis and neural stimulation to improve the management of patients with neurological diseases over the next few years. The articles in the present Research Topic provide a range of material, including novel data and reference resources that will be useful in fostering research in the field and optimizing therapeutic options for patients with movement disorders and brain tumors.

## Author Contributions

All authors equally contributed in the conception, writing, and review of this editorial article.

## Conflict of Interest

The authors declare that the research was conducted in the absence of any commercial or financial relationships that could be construed as a potential conflict of interest.

## Publisher's Note

All claims expressed in this article are solely those of the authors and do not necessarily represent those of their affiliated organizations, or those of the publisher, the editors and the reviewers. Any product that may be evaluated in this article, or claim that may be made by its manufacturer, is not guaranteed or endorsed by the publisher.
